# Using Shopping Data to Improve the Diagnosis of Ovarian Cancer: Computational Analysis of a Web-Based Survey

**DOI:** 10.2196/37141

**Published:** 2023-03-31

**Authors:** Elizabeth H Dolan, James Goulding, Laila J Tata, Alexandra R Lang

**Affiliations:** 1 Neodemographics Lab, Nottingham University Business School, University of Nottingham Nottingham United Kingdom; 2 Lifespan and Population Health School of Medicine University of Nottingham Nottingham United Kingdom; 3 Human Factors Faculty of Engineering University of Nottingham Nottingham United Kingdom

**Keywords:** carcinoma, ovarian epithelial, ovarian neoplasms, self-medication, diagnostic errors, symptom assessment, machine learning, nonprescription drugs, over-the-counter, pharmaceutical, symptom, ovary, ovarian cancer, oncology, cancer

## Abstract

**Background:**

Shopping data can be analyzed using machine learning techniques to study population health. It is unknown if the use of such methods can successfully investigate prediagnosis purchases linked to self-medication of symptoms of ovarian cancer.

**Objective:**

The aims of this study were to gain new domain knowledge from women’s experiences, understand how women’s shopping behavior relates to their pathway to the diagnosis of ovarian cancer, and inform research on computational analysis of shopping data for population health.

**Methods:**

A web-based survey on individuals’ shopping patterns prior to an ovarian cancer diagnosis was analyzed to identify key knowledge about health care purchases. Logistic regression and random forest models were employed to statistically examine how products linked to potential symptoms related to presentation to health care and timing of diagnosis.

**Results:**

Of the 101 women surveyed with ovarian cancer, 58.4% (59/101) bought nonprescription health care products for up to more than a year prior to diagnosis, including pain relief and abdominal products. General practitioner advice was the primary reason for the purchases (23/59, 39%), with 51% (30/59) occurring due to a participant’s doctor believing their health problems were due to a condition other than ovarian cancer. Associations were shown between purchases made because a participant’s doctor believing their health problems were due to a condition other than ovarian cancer and the following variables: health problems for longer than a year prior to diagnosis (odds ratio [OR] 7.33, 95% CI 1.58-33.97), buying health care products for more than 6 months to a year (OR 3.82, 95% CI 1.04-13.98) or for more than a year (OR 7.64, 95% CI 1.38-42.33), and the number of health care product types purchased (OR 1.54, 95% CI 1.13-2.11). Purchasing patterns are shown to be potentially predictive of a participant’s doctor thinking their health problems were due to some condition other than ovarian cancer, with nested cross-validation of random forest classification models achieving an overall in-sample accuracy score of 89.1% and an out-of-sample score of 70.1%.

**Conclusions:**

Women in the survey were 7 times more likely to have had a duration of more than a year of health problems prior to a diagnosis of ovarian cancer if they were self-medicating based on advice from a doctor rather than having made the decision to self-medicate independently. Predictive modelling indicates that women in such situations, who are self-medicating because their doctor believes their health problems may be due to a condition other than ovarian cancer, exhibit distinct shopping behaviors that may be identifiable within purchasing data. Through exploratory research combining women sharing their behaviors prior to diagnosis and computational analysis of these data, this study demonstrates that women’s shopping data could potentially be useful for early ovarian cancer detection.

## Introduction

Ovarian cancer is often diagnosed at an advanced stage, leading to lower 5-year survival rates compared to those for other cancers [1]. When diagnosed at a late stage, 54% of the people survive for a year or more compared to 98% when diagnosed at the earliest stage [1]. Reid et al’s [2] survey of 1531 women with ovarian cancer from 44 countries found that the United Kingdom had the lowest percentage of women (30%) and Italy the highest percentage of women (62.3%) diagnosed with ovarian cancer within 1 month of first visiting a doctor [2].

The reasons for late diagnosis are unclear but may partially be due to symptomatic presentation that is nonspecific and not well-defined clinically [3-5]. The assessment of the shopping behavior for products that may be purchased in reaction to these symptoms represents an approach that could improve the evaluation of prediagnostic delay. Two small-scale studies consisting of 26 interviews [6] and examination of prediagnosis loyalty card data for 6 women [7] have previously provided evidence of individuals self-medicating through health purchases in response to early symptoms of gynecological cancers. How prevalent this behavior is among women with ovarian cancer and why women buy products remain undetermined. However, the potential success of this line of investigation is supported by evidence of self-medication linked to an individual’s pathway to diagnosis relating to patient self-appraisal and self-management of symptoms in the decision to seek help [8]; the frequency drop of general practitioner (GP) consultations and patient self-misdiagnosis [9]; misdiagnosis and masking of symptoms [10]; and delay in seeking health care for rheumatoid arthritis [11], tuberculosis [12], and gastrointestinal cancers [13].

Loyalty card data collect information on customer purchases, such as item type, spending category, purchase amount, time stamp, and store location. This is an area of growing interest, given that the General Data Protection Regulation [14] now gives people the right to obtain their personal data collected by organizations, thus enabling individuals to donate loyalty card data to medical studies [15-18]. Previous studies have also shown that computational analysis of such shopping data, collected through retailers’ loyalty card schemes, in terms of diet and self-medication, are able to produce valuable, new, and previously unavailable insights into population health [19,20]. Set against this background, the objective of this exploratory study was to gain new domain knowledge from women’s experiences, better understand how women’s shopping behavior relates to their pathway to the diagnosis of ovarian cancer, and inform this growing research imperative.

## Methods

### Survey Design

**Figure 1:**
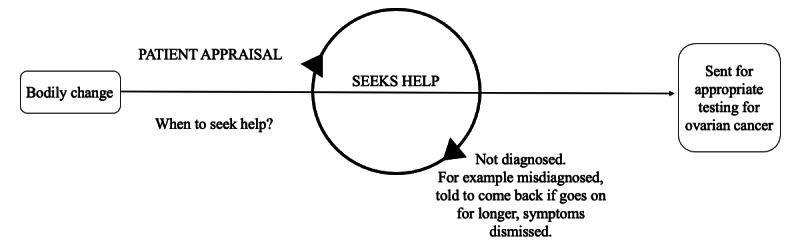
Pathway to ovarian cancer diagnosis. Adapted from Scott et al [8].

A web-based survey study was established to investigate health and shopping patterns in relation to ovarian cancer. The survey was developed by the research team in direct collaboration with Ovacome [21], a UK National Charity that supports around 18,000 people a year affected by ovarian cancer. The survey asked women to report their experience of symptoms and shopping habits for nonprescription health care products prior to their diagnosis with ovarian cancer across a series of 53 questions (Multimedia Appendix 1), divided into the following sections: information on diagnosis; health problems and if, what and why you purchased health products related to them; the impact of health care product purchases; donating loyalty card data; and demographics. Administered via the Jisc online survey tool [22], the survey was designed to elicit knowledge on how shopping behavior interacts with a woman’s pathway to diagnosis, as illustrated in Figure 1 (adapted from Scott et al’s [8] model of pathways to treatment) and with correspondence to the depiction of events prior to a diagnosis of ovarian cancer from Mullins et al [23]. Survey questions were also specifically designed to examine routes to diagnosis (Q11), awareness of symptoms of ovarian cancer (Q12), timings of health problems and health product buying (Q15 and Q22), influence and rationale in the decision-making process to buy health care products (Q17-21), and the impact of buying health care products (Q36-46). Free textboxes also enabled participants to further describe their experience of health care products.

Most questions were optional, and survey data were only stored on completion. Health problems prior to ovarian cancer diagnosis were obtained from Goff et al [24], National Institute for Health and Care Excellence [5], and advised by Ovacome. Health care product types were those that had been identified as likely to be bought in relation to these problems, also advised by Ovacome, with the option to name “Other” types provided to respondents. Products were divided into 12 types, with explanations provided where necessary, and accompanied by photos of example products. Multiple-choice options were decided upon via researcher engagement with women attending Ovacome events and desk research of products available both online and in physical stores.

### Participant Recruitment

The target population of the study was women with a diagnosis of ovarian cancer. Given the fact that recruitment of women with a diagnosis of ovarian cancer is evidenced as challenging [25-27], a pragmatic target of 100 participants was set to underpin this exploratory work. Participants were recruited through Ovacome via their community, including social media sites and web-based health forums. The web-based survey was open from February 23, 2020, to June 3, 2020 (posts advertising the survey are shown in Multimedia Appendix 2 and Multimedia Appendix 3). The survey was distributed via a link to the survey site, where the only content was the survey itself. The survey was open to all, but participants were automatically directed out of the survey if they answered no to “Have you been diagnosed with ovarian cancer?” The informed consent process was delivered through an integrated web-based participant information sheet, privacy notice, and consent form to which participants had to agree before they could complete the survey (See Multimedia Appendix 1).

### Ethics Approval

Ethics approval was obtained from the University of Nottingham (ethics panel reference: CS-2019-R28). Ovacome, the ovarian cancer charity who distributed the survey, agreed to give support to anyone who found the survey upsetting via phone, web chat, or email. The availability of this support was made clear in the participant information.

### Data Analysis

A first-stage descriptive analysis of the data set was performed, with visualizations and derivations from the survey responses being aggregated to establish domain summaries of women’s experiences captured within the data, including what health problems (possible symptoms) women presented with and whether women thought they had conditions other than ovarian cancer. After statistical testing, a logistic regression model was fit to the data to assess odds ratios (ORs) and 95% CIs to examine the following:

1. Whether the duration of health problems reported prior to a diagnosis of ovarian cancer was associated with the purchase of health care products.
2. Whether the duration of health problems reported prior to a diagnosis of ovarian cancer was associated with the purchase of health care products because the participant’s doctor thought their health problems were due to a condition but not ovarian cancer.
3. Whether the duration of buying health care products for health problems reported was associated with the purchase of health care products because a participant’s doctor thought their health problems were due to a condition but not ovarian cancer.
4. Whether the number of health care product types purchased was associated with the purchase of health care products because a participant’s doctor thought their health problems were due to a condition but not ovarian cancer.

Each of the 4 logistic regression models, created to investigate the above, tested the effect of a single independent variable on the categorical dependent variable and were not adjusted models. This method was used to identify potential indicators to use in the exploratory predictive modelling. The analysis was undertaken using the Python Stats model module.

### Exploratory Predictive Modelling

A second-stage predictive analysis was then implemented to explore nonlinear relationships between independent and dependent variables and to examine the potential of using loyalty card data to support predictive inferences about women’s ovarian cancer diagnoses. A machine learning approach was applied with random forest (RF) classifiers (specifically the RandomForestClassifier() from Python’s *scikit-learn* framework) by using a cross-validated grid search. Independent variables used in the modelling process included those shopping data variables (features) whose β values demonstrated statistical significance as identified by the logistic regression analysis in the previous stage (duration of buying and the total amount of product types bought), alongside the counts for each type of product that women purchased (from the top 10 product types bought). Resulting models were then used to assess if purchasing health care products because a participant’s doctor thought their health problems were due to a condition other than ovarian cancer could be predicted (identified) based upon participant buying patterns. A common challenge in modelling using relatively small samples (n=57) is avoidance of overfitting, which can lead to overoptimistic model performance [28]. To attend to this and to assess the generalizability of models on out-of-sample data sets, a rigorous nested k-fold cross-validation (CV inner k-fold=10, CV outer k-fold=10) was further applied [29], generating alternative test data sets from the original data (See Multimedia Appendix 4 for Python code used). The logistic regression model was used to investigate OR (CIs). RF models were used to determine the predictive potential of the data. For reference predictive results from the logistic regression model for the classification of participants using the same inputs as RF models, the accuracy was 77% (fit to all data).

## Results

### Participant Characteristics

The survey was completed by 101 women (Table 1) who had been diagnosed with ovarian cancer between 1996 and 2020 from 12 different regions of the United Kingdom. Most women (92/101, 91.1%) were from White ethnic groups, diagnosed via their GP (68/101, 67.3%) and unaware of the symptoms of ovarian cancer before their diagnosis (71/101, 70.3%). There was a 97.2% (1571/1616) completion rate for the 16 questions that applied to all participants and 97.2% (516/531) completion rate for the 9 questions that applied to participants who bought health care products. Other questions only applied to those participants who carried out a particular behavior (eg, purchasing of a pain relief product).

**Table 1:** Patient demographics and clinical characteristics of the women with a diagnosis of ovarian cancer and their response to health problems and loyalty card use (N=101)^a^.

Characteristic			Values
Age (years), mean (SD)			55.5 (10.69)
Current UK resident, n (%)			95 (94.1)
**Race/ethnicity, n (%)**			
	White	92 (91.1)	
	Asian	5 (5)	
	Black	2 (2)	
	Other	1 (1)	
	Prefer not to say	1 (1)	
**Routes to diagnosis, n (%)**			
	Via a general practitioner	68 (67.3)	
	Other routes	30 (29.7)	
General practitioner appointments, mean (SD)			3.66 (3.29)
**Unaware of the symptoms of ovarian cancer before their diagnosis, n (%)**			
	Yes	71 (70.3)	
	No	27 (26.7)	
**Stage of cancer at diagnosis, n (%)**			
	Unknown	6 (5.9)	
	1	21 (20.8)	
	2	10 (9.9)	
	3	45 (44.6)	
	4	19 (18.8)	
**Reported symptoms matching those given by the NICE^b^ [5] and Goff et al [24] for ovarian cancer, n (%)**			
	Bloating	66 (65.3)	
	Fatigue (tiredness)	58 (57.4)	
	Change in urination habit	55 (54.5)	
	Abdominal pain (tummy pain)	52 (51.5)	
	Change in bowel habit	47 (46.5)	
	Change in appetite	38 (37.6)	
	Indigestion	31 (30.7)	
	Irregular bleeding	28 (27.7)	
	Backache	25 (24.8)	
	Other	21 (20.8)	
	Nausea	19 (18.8)	
	I experienced no health problems	2 (2)	
**In response to the health problems of ovarian cancer prior to diagnosis, n (%)**			
	Bought nonprescription health care products	59 (58.4)	
	Changed their diet	39 (38.6)	
	Bought new clothes	28 (27.7)	
	Exercised	18 (17.8)	
	Other action	13 (12.9)	
Had loyalty cards, n (%)			91 (90.1)
**Most frequently held loyalty cards, n (%)**			
	Boots	73 (72.3)	
	Nectar	66 (65.3)	
	Tesco	64 (63.4)	
Willing to donate their loyalty card data to investigate the diagnosis of ovarian cancer, n (%)			29 (28.7)

^a^Not all values will add up to 101, as there are missing data for some variables.

^b^NICE: National Institute for Health and Care Excellence.

### Women’s Purchases

**Figure 2:**
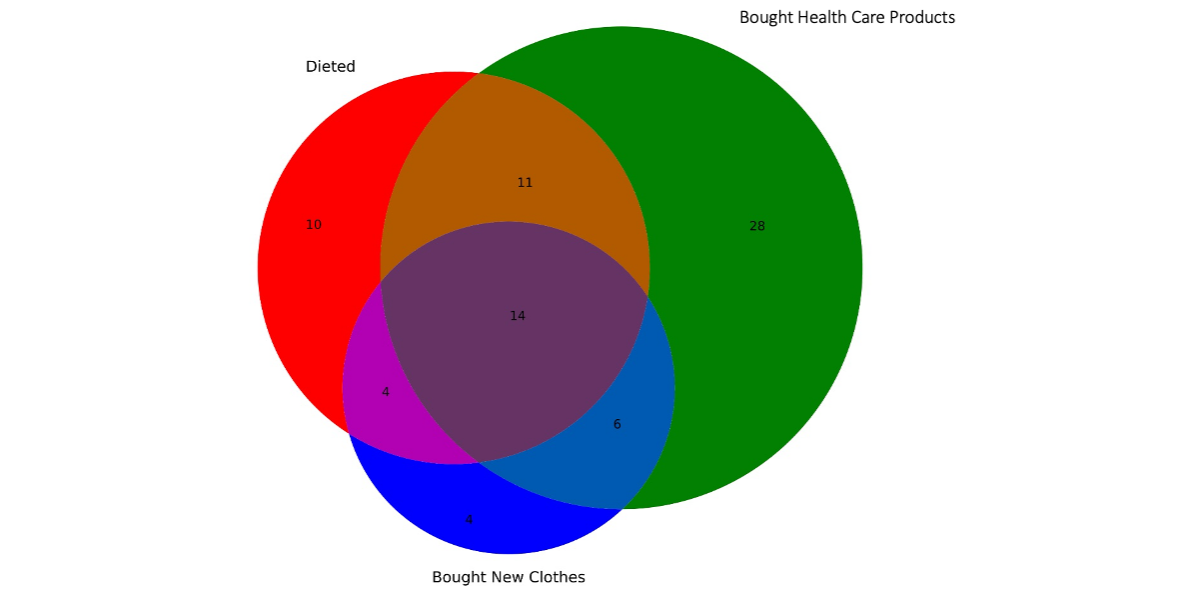
Number of women with ovarian cancer who reported changing their diet and purchasing health care products and new clothes in response to the health problems due to ovarian cancer prior to their diagnosis.

Behaviors related to shopping included change of diet, purchase of nonprescription health care products, and purchase of new clothes (Table 1). Figure 2 shows the number of women who undertook more than one of these behaviors. A wide range of health care product types was purchased (Table 2), with women buying a mean of 3.88 different health care product types in response to the health issues caused by ovarian cancer prior to diagnosis. The product category with the highest increase in purchasing levels was abdominal products, with 76% (45/59) of the women never or rarely purchasing prior to their symptoms. The most purchased health care product (32/59, 54%) out of the 5 types of abdominal products was for trapped wind. Prior to symptoms, a lower proportion of women often or always purchased pain relief (16/59, 27%) and vitamins (6/59, 10%) in comparison to those who bought in response to symptoms (pain relief 38/59, 64%; vitamins 19/59, 32%).

Most health care products (71/102, 69.6%) purchased were reported as ineffective in relieving symptoms (Table 3). This ineffectiveness was confirmed within the qualitative descriptions. For example, *“*Not effective took combination daily was still in a lot of pain;” “Trapped wind products first, then indigestion remedies, then herbal teas, would soothe symptoms for a while but they always came back, so I’d return to the GP.”

**Table 2:** Purchasing nonprescription health care products prior to ovarian cancer diagnosis and clinical influences to buy (n=59).

Variable				Values
Health care product types purchased in response to the health problems of ovarian cancer prior to diagnosis, mean (SD)				3.88 (2.13)
**Health care product types purchased, n (%)**				
	Pain relief product			38 (64)
	Trapped wind product			32 (54)
	Irritable bowel syndrome products			23 (39)
	Incontinence or period products			23 (39)
	Constipation product			19 (32)
	Vitamins			19 (32)
	Wheat bags, heat pads, or hot water bottles			17 (29)
	Gut health products			16 (27)
	Pain relief with codeine			15 (25)
	Under eye cream and concealer products			13 (22)
	Diarrhea product			9 (15)
	Cystitis relief products			5 (8)
**Purchasing of health care products before the health problems of ovarian cancer, n (%)**				
	**Pain** **relief**			
		Never or rarely	24 (41)	
		Sometimes	16 (27)	
		Often or always	16 (27)	
	**Abdominal products**			
		Never or rarely	45 (76)	
		Sometimes	6 (10)	
		Often or always	5 (8)	
	**Vitamins/supplement products**			
		Never or rarely	38 (64)	
		Sometimes	11 (19)	
		Often or always	6 (10)	
Purchased health care products because they suspected they had a specific condition that was not ovarian cancer, n (%)				44 (75)
**Condition other than ovarian cancer, n (%)**				
	Diarrhea			25 (42)
	Indigestion problems such as stomachache			25 (42)
	Constipation			25 (42)
	Heartburn			25 (42)
Purchased health care products because their doctor thought their health problems were due to a condition other than ovarian cancer, n (%)				30 (51)
**Conditions frequently suspected by doctors, n (%)**				
	Irritable bowel syndrome			10 (17)
	Diverticulitis			4 (7)
	Menopause			4 (7)
	Constipation			4 (7)
Prescribed medication because their doctor thought health problems were due to a condition other than ovarian cancer, n (%)				24 (41)
**Prescriptions frequently given by doctors, n (%)**				
	Irritable bowel syndrome medication			5 (8)
	Laxatives			5 (8)
	Antibiotics			4 (7)
	Medication for reflex			4 (7)

**Table 3:** Nonprescription health care products purchased prior to ovarian cancer diagnosis and the time taken to see if they would work.

				Values, n (%)
**Time waited to see if health care products work**				
	**Abdominal products (n=44)**			
		Two weeks or longer	20 (45)	
		A month or longer	15 (34)	
	**Vitamins/supplements (n=20)**			
		A month or longer	17 (85)	
		Longer than a month	12 (60)	
**Products did not work or only worked for a few hours**				
	Pain relief (n=38)			27 (71)
	Abdominal products (n=44)			30 (68)
	Vitamins/supplements (n=20)			14 (70)

### Why Women Purchased Health Care Products

Advice from your GP was the top answer respondents provided when asked what influenced their purchase of nonprescription health care products (23/59, 39%), followed by advice from friends and family (18/59, 31%) and advice found on websites (15/59, 25%). The survey identified that most women (44/59, 75%) were motivated to buy health care products because they suspected they had a specific condition that was not ovarian cancer. Of women who purchased health care products, 51% (30/59) bought nonprescription health care products specifically because their doctor had thought their health problems were due to a condition other than ovarian cancer. Many women (24/59, 41%) who bought health care products were also supplied with prescription medication due to their doctor believing health problems were due to a condition other than ovarian cancer.

### Waiting to See If Health Care Products Work

Of participants who bought abdominal health care products prior to diagnosis of ovarian cancer, 45% (20/44) waited 2 weeks or more to see if they worked and 34% (15/44) waited a month or more. Although fewer women bought vitamins or supplements, a larger percentage (17/20, 85%) waited a month or longer to see if they would prove effective.

### Loyalty Card Data Donation

The majority of the women (91/101, 90.1%) in the survey had loyalty cards with 72.3% (73/101), 65.3% (66/101), and 63.4% (64/101) having cards from Boots, Nectar, and Tesco, respectively—the 3 top retailers in the United Kingdom—and 28.7% (29/101) of the women gave contact details to share their loyalty card data. Respondents filtered themselves out of giving loyalty card data if they had not used loyalty cards often, their data were old/out-of-date, or they had not made purchases. For example, “I don’t think my loyalty data is relevant becoz I didn’t buy any off the shelf medications. But if you still feel it’s relevant to your research, contact me.”

### Relationships Between Health Care Product Purchases and Ovarian Cancer Diagnosis Pathway

**Figure 3:**
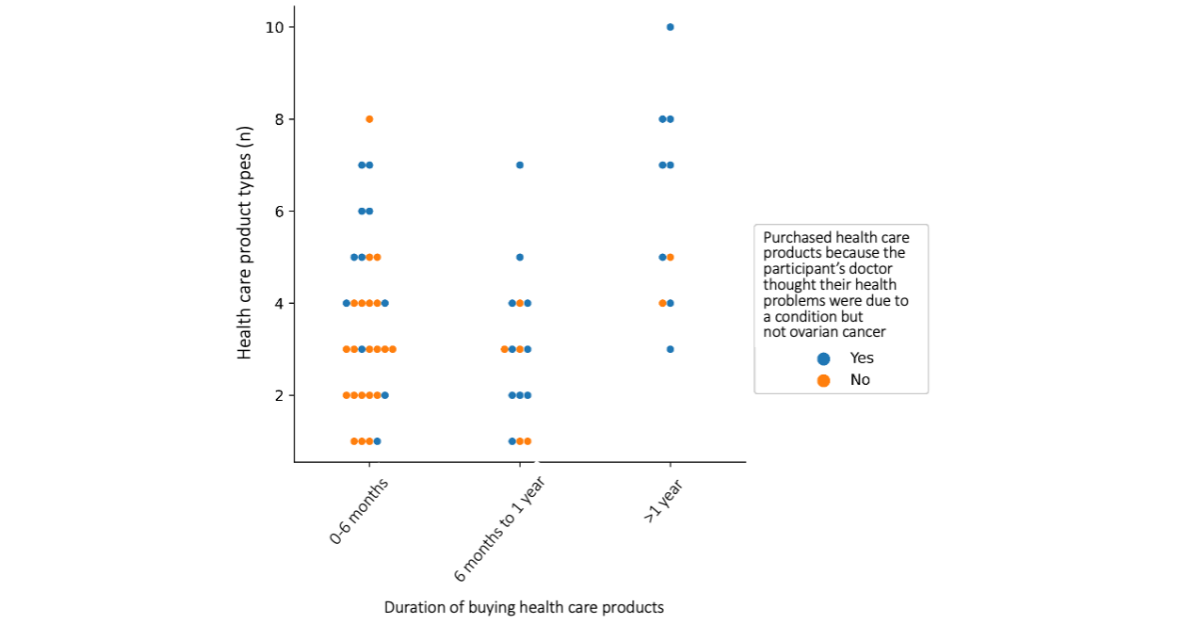
Categorical scatterplot comparing the duration of buying and number of different health care product types purchased by women with ovarian cancer because their doctor thought their health problems were due to a condition but not ovarian cancer.

**Figure 4:**
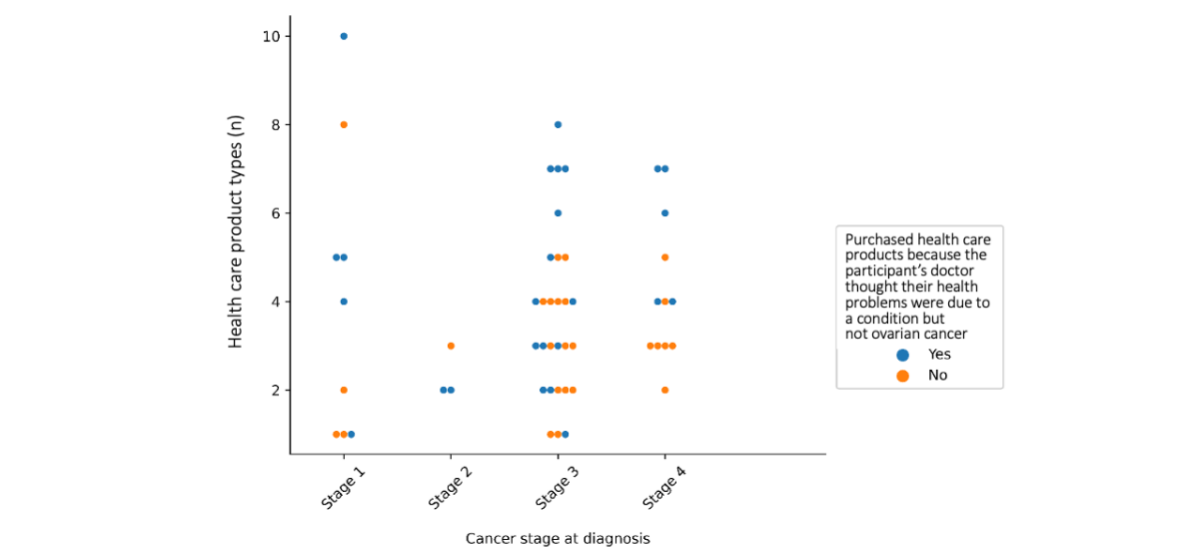
Categorical scatterplot comparing cancer stage at diagnosis and number of different health care product types purchased by women with ovarian cancer because their doctor thought their health problems were due to a condition but not ovarian cancer.

Figure 3 illustrates both the number of product types women bought and their duration of buying health care products. Plotting both these variables reveals an observable difference in the purchasing patterns in women who self-medicated because their doctor thought their health problems were due to a condition other than ovarian cancer. Figure 4 illustrates the number of product types women brought and the stage of cancer at diagnosis. It indicates woman are more likely to be shopping as a result of doctor’s advice that their health problems were due to a condition other than ovarian cancer when they have purchased 6 or more health care product types. However, only 23% (12/52) of the women surveyed, who reported the stage of cancer at diagnosis and bought health care products, were in an early enough stage (stage 1 or 2) of cancer at diagnosis to draw reliable results about the relationship between cancer stage and their purchasing patterns.

Women who bought health care products were no more likely to have had a longer duration of health problems prior to a diagnosis of ovarian cancer (Table 4). When considering only those participants who purchased health care products, women were 7 times more likely to have had a duration of more than a year of health problems prior to a diagnosis of ovarian cancer (Table 5) if they were self-medicating based on advice from a doctor, rather than having made the decision to self-medicate independently (OR 7.33, 95% CI 1.58-33.97). Women in this situation, who were making purchases due to their doctor believing their health problems may be due to a condition other than ovarian cancer, were more likely to have shopped for 6 months to a year (OR 3.82, 95% CI 1.04-13.98) or more than a year (OR 7.64, 95% CI 1.38-42.33) (Table 6). The likelihood that a participant was shopping because their doctor thought their health problems were due to some condition other than ovarian cancer increased with every extra product type they purchased (OR 1.54, 95% CI 1.13-2.11). Multimedia Appendix 5 shows the distribution of the different product types purchased.

**Table 4:** Results from the logistic regression model on the relationship between the duration of the health problems prior to the diagnosis of ovarian cancer and participant purchasing of health care products.

Duration of health problems	Participant purchasing of health care products				
	No (n=42), n (%)	Yes (n=59), n (%)	Total (N=101)	Odds ratio (95% CI)	*P* value
<6 months	23 (55)	25 (42)	48	Reference	N/A^a^
6 months-1 year	10 (24)	20 (34)	30	1.84 (0.71-4.74)	.21
>1 year	9 (21)	14 (24)	23	1.43 (0.52-3.93)	.49

^a^N/A: Not applicable.

**Table 5:** Results from the logistic regression model on the relationship between the duration of health problems prior to the diagnosis of ovarian cancer and participant purchasing of health care products because their doctor thought their health problems were due to a condition but not ovarian cancer.

Duration of health problems	Bought health care products because their doctor thought their health problems were due to a condition but not ovarian cancer				
	No (n=28), n (%)	Yes (n=30), n (%)	Total (n=58)	Odds ratio (95% CI)	*P* value
<6 months	16 (57)	8 (27)	24	Reference	N/A^a^
6 months-1 year	9 (32)	11 (37)	20	2.44 (0.72-8.31)	.15
>1 year	3 (11)	11 (37)	14	7.33 (1.58-33.97)	.01

^a^N/A: Not applicable.

**Table 6:** Results from the logistic regression model on the relationship between the duration of buying health care products prior to the diagnosis of ovarian cancer and participant purchasing of health care products because their doctor thought their health problems were due to a condition but not ovarian cancer.

Duration of buying	Bought health care products because their doctor thought their health problems were due to a condition but not ovarian cancer				
	No (n=28), n (%)	Yes (n=29), n (%)	Total (n=57)	Odds ratio (95% CI)	*P* value
<6 months	21 (75)	11 (38)	32	Reference	N/A^a^
6 months-1 year	5 (18)	10 (34)	15	3.82 (1.04-13.98)	.04
>1 year	2 (7)	8 (28)	10	7.64 (1.38-42.33)	.02

^a^N/A: Not applicable.

### Exploring Predictive Capabilities of Purchasing Data

**Figure 5:**
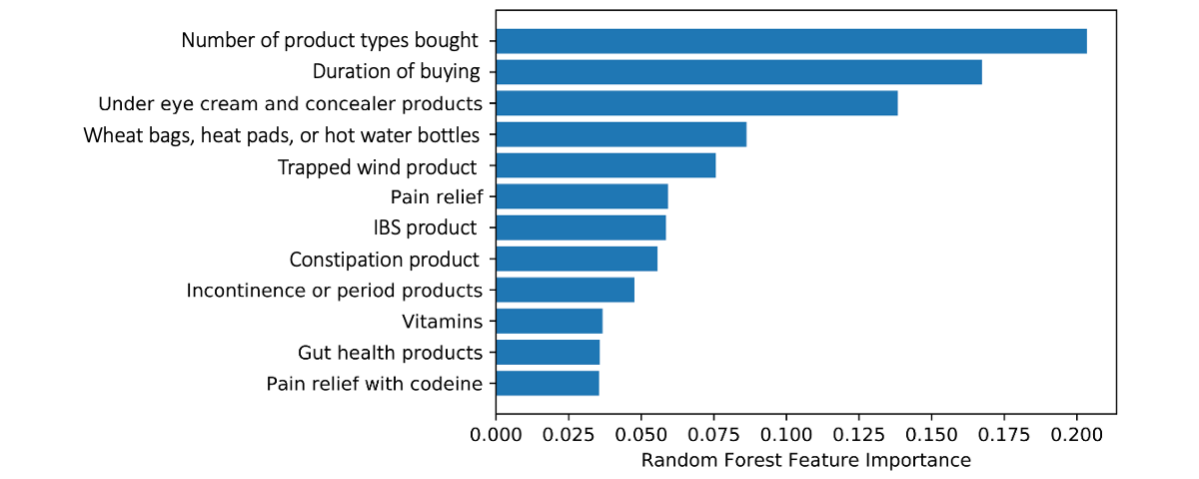
Bar chart comparing random forest variable (feature) importance. IBS: irritable bowel syndrome.

Optimized RF models were able to correctly predict the class of 25 out of 29 women who had been shopping because their doctor thought their health problems were due to a condition other than ovarian cancer (with 4 false negatives) and 26 out of the 28 who had chosen to self-medicate independently (with 2 false positives). On average, RF modelling produced classifiers with an accuracy score of 89.1%, a recall score of 89.1%, and a precision score of 89.8% (average scores from 10 RF models). Figure 5 plots the variable (feature) importance revealed by the modelling process. To assess generalizability of the models on out-of-sample data, nested k-fold CV (CV inner k-fold=10, CV outer k-fold=10) was implemented for each of the 3 assessment scores considered (classification accuracy/precision/recall). Due to the stochastic nature of nested CV, 10 experimental runs were implemented using different random seeds each time. The mean scores across all experimental runs returned an average classification accuracy score of 70.1% (SD 20%), an average precision score of 76.4% (SD 26.8%), and an average recall score of 77.9% (SD 23.7%).

## Discussion

### Principal Findings

Our study is the first to evidence how women change their shopping habits in response to the health problems caused by ovarian cancer prior to a diagnosis. The majority of women (59/101, 58.4%) bought nonprescription health care products in response to symptoms, most being for pain relief (38/59, 64%), followed by abdominal ailments, incontinence, bleeding, and fatigue. Women in the survey were 7 times more likely to have had a duration of more than a year of health problems prior to a diagnosis of ovarian cancer if they were self-medicating based on advice from a doctor, rather than having made the decision to self-medicate independently. Our results also show that women waited for several weeks or longer to see if health care products reduced their symptoms, with advice from the GP being the top influence for purchasing health care products. This study indicates that increased shopping for health care products is associated with cases where women are receiving advice from a doctor who believe their health problems are due to a condition other than ovarian cancer. Further investigation is required to determine if receiving such advice from a doctor might disproportionately increase the time women self-manage symptoms prior to reseeking help, leading to a longer duration to an accurate diagnosis—especially given that the diagnosis of ovarian cancer often occurs at a late stage [1] and doctors in the United Kingdom take longer to refer patients for appropriate investigations compared to doctors in other western countries [2].

### Comparison With Prior Work

The study corroborates the findings of previous studies with smaller sample sizes [6,7] by showing the prevalence of self-medication strategies in women with ovarian cancer. The results of our study and the methodologies discussed could be applied to investigate different diseases. Other research reports delay to diagnosis due to self-medication for other conditions [11-13]; however, the reasons for participants self-medicating remained unexplained. Specific buying behaviors reported in these studies varied by disease. For rheumatoid arthritis in the United Kingdom, patients bought tablets from the chemist, but with few speaking to pharmacists [11], and for gastrointestinal cancer in Nepal, patients used alternative medicines and antacids [13]. The increased median time between the onset of symptoms and diagnosis associated with self-medication also varied in these studies from 2.2 weeks for rheumatoid arthritis [11] to over 17 weeks for gastrointestinal cancer [13]. Unlike the results reported in this study, previous studies did not explore in as much granularity the specific health care products that participants bought. A comparison of the buying patterns of women with ovarian cancer examined in this study with those examined in previous research indicates that buying patterns likely vary between different diseases and geographical environments, both in product type and timings of purchases. Finally, almost a third of women surveyed reported that they would be willing to provide access to their loyalty card data to assist a next-stage study. Previous studies have demonstrated that willingness to share loyalty card data varies according to several factors [17,18], and this has been further demonstrated by the qualitative data provided by the women in our survey.

### Limitations in This Study

This study did not look at the shopping habits of women without ovarian cancer. It therefore remains an open research question as to whether identifiable differences in shopping behaviors can be found between women who developed ovarian cancer and those who did not [30]. As an exploratory and hypothesis-generating approach, no causality can be inferred from our study. Despite the recruitment process occurring in partnership with Ovacome, due to the use of an open web-based survey, women’s ovarian cancer was self-declared rather than clinically confirmed. The shopping data collected were reliant on women’s memories and ability to recall correctly, and the study sample is not representative of the population of women diagnosed with ovarian cancer in the United Kingdom. Recruitment exclusively via the Ovacome community may have also led to other sample bias; the average age of the participants was 55.5 (SD 10.69) years, whereas ovarian cancer incidence rates in the United Kingdom are the highest in females aged 75 to 79 years [1]. The terminology “health problems” was used to ask women about symptoms prior to their diagnosis of ovarian cancer, as women may not have realized these were symptoms. However, it may mean that coincidental health problems have been considered. Although the sample size in our study was notably larger than that in previous studies conducted in this field [6,7], the sample size was still small.

### Conclusions

Through exploratory research, our study demonstrates that analysis of information collected on women’s shopping data may potentially be useful for early ovarian cancer detection. Future studies using loyalty card data could provide accurate information on patients’ behavior and symptoms between consultations where medical data are currently not available. This could be used to investigate what can influence and delay patient help-seeking. Advances in using loyalty card data for health research, made possible due to novel machine learning techniques [19,20], raise the question: Could carefully applied modelling of shopping data be a useful tool in investigating the diagnosis of and expression of symptoms in diseases such as ovarian cancer? This study confirms the importance of consulting with the patient stakeholder to “choose the right problem to address” before considering using machine learning in health care [31]. This study provides evidence that a distinctive pattern in shopping for health care products could be associated with the purchase of health care products because a participant’s doctor thought their health problems were due to a condition but not ovarian cancer. The RF models, derived from the knowledge and data obtained from the survey, represent an exploratory modelling approach constructed from a limited sample size. However, with an out-of-sample classification accuracy of 70.1% and recall of 77.9% showing a capability for high sensitivity, they serve to demonstrate the potential to use machine learning to identify women with later diagnosis or a higher risk of a longer duration to an accurate diagnosis of ovarian cancer by using big data sets collected via loyalty cards.

An analysis of loyalty card data could provide evidence to support and enhance women’s self-reported narratives. Further studies using loyalty card data could profitably be carried out to establish the precise periods women are waiting to assess the effectiveness of health care products and the exact time delay to diagnosis purchasing health care products can cause. If an analysis of loyalty card data confirmed the findings from this study, it would not only provide probabilistic insight at a national level but also provide evidence to invest in the development of the following 3 initiatives. First, advice on guidelines to doctors and GPs about the recommendation of self-medication when dealing with the following symptoms in women: bloating, feeling full/loss of appetite, pelvic or abdominal pain, increased urinary urgency/frequency, weight loss, fatigue, and change in bowel movements [5]—especially in terms of the ineffectiveness of self-medication for women with ovarian cancer and the critical time delay the recommendation of self-medication can cause. Second, pharmacists in retail settings could observe shoppers whose purchasing appears to follow the discovered pattern from the loyalty card data analysis, and with an individual’s permission, assess if they require further investigations for ovarian cancer. Pharmacists could also consider prescription data, as 41% (24/41) of the women with ovarian cancer who bought health care products were also given a prescription because their doctor thought their health problems were related to a condition but not ovarian cancer. Third, a new clinical tool could be developed to identify women with ovarian cancer, which includes asking them about their purchasing habits. This could be implemented by GPs, doctors in accident and emergency departments, and pharmacists.

## Appendix

Multimedia Appendix 1 Web-based survey.

Multimedia Appendix 2 Web-based post advertising the survey on My Ovacome.

Multimedia Appendix 3 Web-based post advertising the survey on Facebook.

Multimedia Appendix 4 Python code used for the machine learning analysis.

Multimedia Appendix 5 Bar chart comparing the health care product types purchased by women because their doctor thought their health problems were due to a condition but not ovarian cancer.

## Abbreviations

CV: cross-validation
GP: general practitioner
OR: odds ratio
RF: random forest
