# Supplementary material for: Using Shopping Data to Improve the Diagnosis of Ovarian Cancer: Computational Analysis of a Web-Based Survey
Source: JMIR Cancer. 2023 Mar 31;9:e37141. doi: 10.2196/37141 (PMC10131768; doi:10.2196/37141)
Supplement: Multimedia Appendix 1 [file cancer_v9i1e37141_app1.pdf]

This survey is part of a research project which aims to get women diagnosed earlier. The research is looking at possible links between women's healthcare shopping habits while they were experiencing possible early symptoms and the diagnosis of ovarian cancer. You can help us by taking part in this survey and telling us your experience of symptoms before being diagnosed with ovarian cancer.

This is how we plan to do it…

- **First**, your participation in this survey can help us establish how many women with undiagnosed ovarian cancer are responding to their health problems with shopping for healthcare products, and why.
- **Second**, the knowledge gained from this survey will then be applied to inform a machine learning *(a method of programming computers to learn from data)* analysis of women's loyalty card data *(customer shopping information held by a retailer)*.
- **Third,** we will use this analysis in order to expand our knowledge about the symptoms of ovarian cancer. For example, the timeframes they occur in, the order, and how women respond to them both physically and behaviourally.
- **Finally**, we will then use this knowledge to help women and Health Care Practitioners recognise the signs of ovarian cancer for earlier diagnosis.

What participation involves...

You fill in an online questionnaire consisting of a series of questions, mainly multiple choice, divided into the following parts:

- Information on diagnosis
- Health problems and if, what and why you purchased health products
- The impact of healthcare product purchases
- Donating loyalty card data
- Demographics

It takes approximately 20-30 minutes to complete the questionnaire.

You are eligible to take part in this survey if you have a diagnosis of ovarian cancer

Participation in the research is completely voluntary, you may choose not to answer any questions, and to stop at any time, without the need to give a reason. You may withdraw from participating at any time, simply by closing your browser window. If you withdraw your participation, the data you have provided so far will NOT be stored or analysed. If at a later time, you inform the researchers that you wish to withdraw your data from the study, any data held that you can be identified from will be deleted or destroyed.

Due to the sensitive nature of the topic covered by the questionnaire there is the risk you may find completing it upsetting. Ovacome, who are assisting us with this survey, offer support for anyone affected by ovarian cancer. You can contact them via:

- 0800 008 7054 (open 10am-5pm Monday-Friday, 10am to 8pm on Tuesdays)
- Chat with them online https://www.ovacome.org.uk
- Or email support@ovacome.org.uk

Why we collect your personal data. We collect personal data under the terms of the University's Royal Charter in our capacity as a teaching and research body to advance education and learning. Specific purposes for data collection on this occasion are for a research project on diagnosing disease with shopping data.

The legal basis for processing your personal data under GDPR. Under the General Data Protection Regulation, the University must establish a legal basis for processing your personal data and communicate this to you. The legal basis for processing your personal data on this occasion is Article 6(1e) processing is necessary for the performance of a task carried out in the public interest.

Special category personal data In addition to the legal basis for processing your personal data, the University must meet a further basis when processing any special category data, including: personal data revealing racial or ethnic origin, political opinions, religious or philosophical beliefs, or trade union membership, and the processing of genetic data, biometric data for the purpose of uniquely identifying a natural person, data concerning health or data concerning a natural person's sex life or sexual orientation.

The basis for processing your sensitive personal data on this occasion is Article 9(2a) the data

subject has given explicit consent to the processing and Article 9(2j) processing is necessary for archiving purposes in the public interest, scientific or historical research purposes or statistical purposes.

How long we keep your data. The University may store your data for up to 25 years and for a period of no less than 7 years after the research project finishes. The researchers who gathered or processed the data may also store the data indefinitely and reuse it in future research.

Who we share your data with. Extracts of your data may be disclosed in published works that are posted online for use by the scientific community. Your data may also be stored indefinitely by members of the researcher team and/or be stored on external data repositories (e.g., the UK Data Archive) and be further processed for archiving purposes in the public interest, or for historical, scientific or statistical purposes.

How we keep your data safe. We keep your data securely and put measures in place to safeguard it. These safeguards include anonymization of data and encryption of devices on which your data is stored.

Your rights as a data subject. GDPR provides you, as a data subject, with a number of rights in relation to your personal data. Subject to some exemptions, you have the right to:

- withdraw your consent at any time where that is the legal basis of our processing, and in such circumstances, you are not obliged to provide personal data for our research.
- object to automated decision-making, to contest the decision, and to obtain human intervention from the controller.
- access (i.e., receive a copy of) your personal data that we are processing together with information about the purposes of processing, the categories of personal data concerned, recipients/categories of recipient, retention periods, safeguards for any overseas transfers, and information about your rights.
- have inaccuracies in the personal data that we hold about you rectified and, depending on the purposes for which your data is processed, to have personal incomplete data completed
- be forgotten, i.e., to have your personal data erased where it is no longer needed, you withdraw consent and there is no other legal basis for processing your personal data, or you object to the processing and there is no overriding legitimate ground for that processing.
- in certain circumstances, request that the processing of your personal data be restricted, e.g., pending verification where you are contesting its accuracy, or you have objected to the processing.
- obtain a copy of your personal data which you have provided to the University in a structured, commonly used electronic form (portability), and to object to certain processing activities such as processing based on the University’s or someone else’s legitimate interests, processing in the public interest or for direct marketing purposes. In the case of objections based on the latter, the University is obliged to cease processing.
- complain to the Information Commissioner’s Office about the way we process your personal data.

Consent form

To participate in this survey, please read the consent form information and select the 'Yes' option to indicate that you accept the following study conditions. Please be aware if you select 'No' to the conditions of the study, you will not be able to continue with the survey.

Taking part in the study

1. I have read the project information page that preceded this page, or it has been read to me, and I have understood it. I consent voluntarily to be a participant in this study and understand that I can refuse to answer questions and I can withdraw from the study at any time, without having to give a reason. I understand that because of the sensitive nature of the subject involved there is a potential risk I may find taking part in the study upsetting, and am aware of the support available. I understand that taking part in the study requires me to provide data and that this will involve responding to questions on this online survey. ✱ *Required*

- Yes
- No (Selecting 'No' means you will not be able to continue with the survey)

Consent form

Use of my data in the study.

2. I understand that data which can identify me will not be shared beyond the project team. I agree that the data provided by me may be used for the following purposes: Presentation and discussion of the project and its results in research activities (e.g., in supervision sessions, project meetings, conferences). Publications and reports describing the project and its results. Dissemination of the project and its results, including publication of data on web pages and databases. I give permission for my words to be quoted for the purposes described above. I understand that my identity is not collected for the purposes of this online survey. I will not be personally identified by my survey responses. If at a later time, I inform the researchers that I wish to withdraw my data from the study, any data held that I can be identified from will be deleted or destroyed.

- Yes
- No (Selecting 'No' means you will not be able to continue with the survey)

Consent form

Reuse of my data

3. I give permission for the data that I provide to be reused for the sole purposes of future research and learning. I understand and agree that this may involve depositing my data in a data repository, which may be accessed by other researchers.

- ○ Yes
- ○ No (Selecting 'No' means you will not be able to continue with the survey)

Consent form

Security of my data

4. I understand that safeguards will be put in place to protect my identity and my data during the research, and if my data is kept for future use. I confirm that a copy of these safeguards has been provided to me in the University’s Privacy Notice and that they are acceptable to me. I understand that no computer system is completely secure and that there is a risk that a third party could obtain a copy of my data.

- Yes
- No (Selecting 'No' means you will not be able to continue with the survey)

Consent form

Copyright

5. I give permission for data gathered during this project to be used, copied, excerpted, annotated, displayed and distributed for the purposes to which I have consented.

- Yes
- No (Selecting 'No' means you will not be able to continue with the survey)

Consent form

6. Do you consent to participate in this online survey? To proceed to the survey questions you must answer YES to this question. You may withdraw from the study at any point by leaving the survey's webpage. ✱ *Required*

- ◯ Yes
- ◯ No (Selecting 'No' means you will not be able to continue with the survey)

Information on diagnosis

7. Have you been diagnosed with ovarian cancer? ✱ *Required*

- Yes
- No

Information on ovarian cancer diagnosis

8. When were you first diagnosed with ovarian cancer? If you do not remember please answer question 3a instead.

Dates need to be in the format 'DD/MM/YYYY', for example 27/03/1980.

(dd/mm/yyyy)

8.a. If you do not remember the exact date please put an estimate.

Dates need to be in the format 'DD/MM/YYYY', for example 27/03/1980.

(dd/mm/yyyy)

9. At what point was the ovarian cancer diagnosed?

- Stage 1
- Stage 2
- Stage 3
- Stage 4
- I do not know

10. Do you know the type of ovarian cancer you have?

- No
- Yes

10.a. If Yes, please specify:

11. How many GP appointments do you remember having before investigations were started for ovarian cancer? Investigations tend to be a blood test (CA125 test) and/or a scan (abdominal or transvaginal ultrasound).

- 1
- 2
- 3
- 4
- 5
- 6
- 7
- 8
- 9
- 10 +
- I first presented my symptoms at A&E (Accident and Emergency) and investigations were carried out there
- My ovarian cancer was discovered because I was being treated for another health problem in hospital. Please give details below.
- Other. Please give details below.

11.a. If you wish to clarify or add additional information on this question please do so here

12. Were you aware of the symptoms of ovarian cancer before your diagnosis?

- Yes
- No

12.a. If you wish to clarify or add additional information on this question please do so here

Addressing health problems prior to diagnosis

13. Please select below any health problems you experienced before being diagnosed with ovarian cancer.

- ☐ Bloating
- ☐ Abdominal Pain (Tummy Pain)
- ☐ Change in urination habit
- ☐ Change in bowel habit
- ☐ Change in appetite
- ☐ Fatigue (Tiredness)
- ☐ Irregular bleeding
- ☐ Indigestion
- ☐ Backache
- ☐ Nausea
- ☐ I experienced no health problems
- ☐ Other

13.a. If you selected Other, please specify:

14. In response to these health problems did you do any of the following:

- ☐ Buy non-prescription healthcare products (including pain relief and other medications)
- ☐ Change your diet
- ☐ Exercise
- ☐ Buy new clothes
- ☐ Other

14.a. If you selected Other, please specify:

14.b. If you wish to clarify or add additional information on this question please do so here

15. Overall, how long do you recall having health problems before your diagnosis?

- A few days
- 1 week
- 2 weeks
- 3 weeks
- 1 month
- 2 - 3 months
- 4 - 5 months
- 6 - 7 months
- 8 - 9 months
- 10 -11 months
- A year
- More than a year

Pictures of example non-prescription healthcare products.

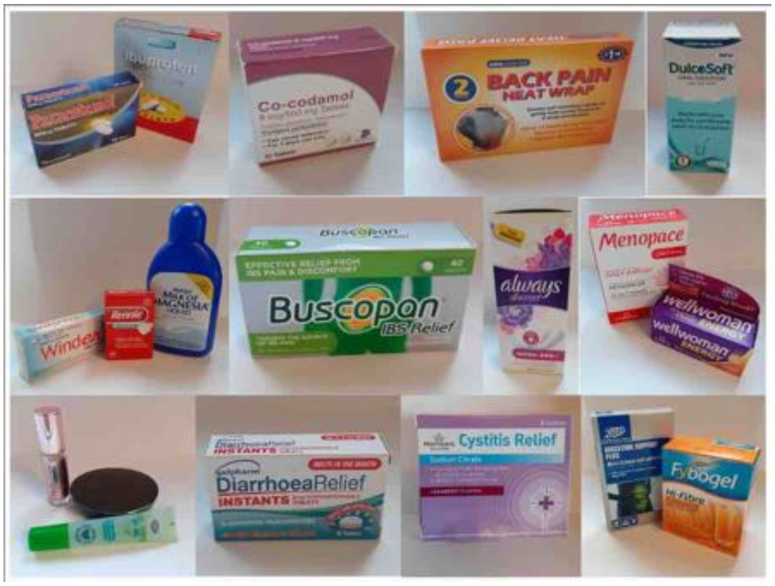

16. Please select an option:

- ◯ I bought non-prescription healthcare products in response to health problems I was having before I was diagnosed with ovarian cancer.
- ◯ I did NOT buy non-prescription healthcare products in response to health problems I was having before I was diagnosed with ovarian cancer.

Addressing health problems in ovarian cancer by shopping

The following section asks questions about your healthcare shopping habits prior to an ovarian cancer diagnosis. Please answer as best as you can. If you do not recall just move onto the next question.

17. Did any of the following influence your decision to buy non-prescription healthcare products in response to your health problems? Please select whose advice you sought first of all, then second, then third... You do not need to use all 7 advice sources, choose as many as you used.

Please don't select more than 1 answer(s) per row.

	1st	2nd	3rd	4th	5th	6th	7th
Advice from friends and family	☐	☐	☐	☐	☐	☐	☐
Advice found on websites	☐	☐	☐	☐	☐	☐	☐
Advice found on social media and online forums	☐	☐	☐	☐	☐	☐	☐
Advice found from NHS material (online or paper e.g. leaflet, poster)	☐	☐	☐	☐	☐	☐	☐
Advice from your GP	☐	☐	☐	☐	☐	☐	☐
Advice from a pharmacist	☐	☐	☐	☐	☐	☐	☐
Advice in advertisements of the products	☐	☐	☐	☐	☐	☐	☐

17.a. Please use this box to describe other influences, or to expand or clarify the answers above if you wish

17.b. If you did not seek advice from anywhere please write why in the box below?

18. Please read the **Key for question 12** below carefully for the descriptions of 0-3 ratings. Please select 0, 1, 2, or 3 to describe when you first bought a healthcare product for the health problem listed in the table.

Please don't select more than 1 answer(s) per row.

	0	1	2	3
Bloating	☐	☐	☐	☐
Abdominal Pain (Tummy Pain)	☐	☐	☐	☐
Change in urination habit	☐	☐	☐	☐
Change in bowel habit	☐	☐	☐	☐
Change in appetite	☐	☐	☐	☐
Fatigue (Tiredness)	☐	☐	☐	☐
Irregular bleeding	☐	☐	☐	☐
Indigestion	☐	☐	☐	☐
Backache	☐	☐	☐	☐
Nausea	☐	☐	☐	☐

Key for question 12:

0 = I did not buy products for this health problem

1 = When I first bought a product for this health problem I did not think I needed to consult a healthcare professional.

2 = When I first bought a product for this health problem I did think I also needed to consult a healthcare professional.

3 = When I first bought a product for this health problem it caused me to immediately consult a healthcare professional.

19. Did you buy non-prescription healthcare products because you thought your health problems meant you had a specific condition?

- ◯ Yes
- ◯ No

19.a. If yes, which condition/s did you attribute them to:

- ☐ Stress/Anxiety
- ☐ IBS
- ☐ Menopause
- ☐ Urinary Tract Infection (UTI)
- ☐ Ageing
- ☐ Hormone Imbalance
- ☐ Muscle / Joint / Sport Injury
- ☐ Hernia
- ☐ Pre-menstrual Syndrome (PMS)
- ☐ Irregular Periods
- ☐ Incontinence
- ☐ Indigestive problems such as stomach ache, constipation, diarrhoea, heartburn
- ☐ Other, please specify

19.a.i. If you selected Other, please specify:

20. Did you buy non-prescription healthcare products because your doctor thought your health problems were due to a condition but not ovarian cancer?

- Yes
- No

20.a. If yes, please state the condition/s *Optional*

21. Did your doctor prescribe medicines because they thought your health problems were related to a condition but not ovarian cancer?

- Yes
- No

21.a. If yes, please state the condition/s and prescribed item/s *Optional*

22. Overall, how long do you recall buying healthcare products for health problems you later realised had been symptoms of ovarian cancer?

- A few days
- 1 week
- 2 weeks
- 3 weeks
- 1 month

- 2 - 3 months
- 4 - 5 months
- 6 - 7 months
- 8 - 9 months
- 10 - 11 months
- A year
- More than a year

Addressing health problems in ovarian cancer by shopping.

Types of Items Purchased

Select which healthcare products you bought for which health problems.

You can select more than one health problem for a product bought. For example, bloating and abdominal pain.

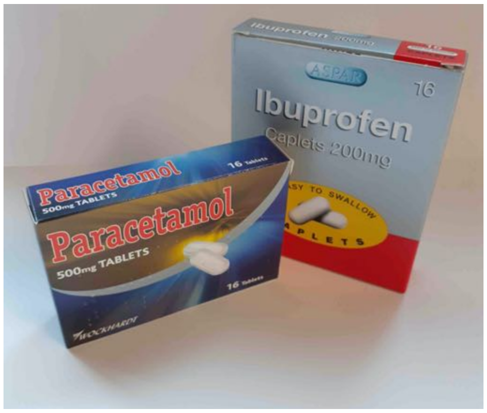

23. **Pain relief** including ibuprofen, paracetamol, aspirin, targeted pain relief product for back and muscle pain or period abdominal pain

- ☐ Bloating
- ☐ Abdominal Pain (Tummy Pain)
- ☐ Change in urination habit
- ☐ Change in bowel habit
- ☐ Change in appetite
- ☐ Fatigue (Tiredness)
- ☐ Irregular bleeding
- ☐ Indigestion
- ☐ Backache
- ☐ Nausea
- ☐ I did not purchase
- ☐ Other, please specify

23.a. If you selected Other, please specify:

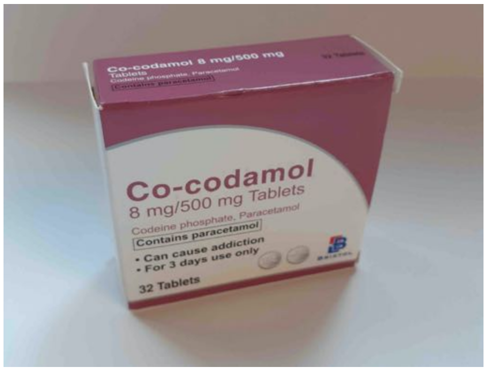

24. **Pain relief with codeine**

- ☐ Bloating
- ☐ Abdominal Pain (Tummy Pain)
- ☐ Change in urination habit
- ☐ Change in bowel habit
- ☐ Change in appetite
- ☐ Fatigue (Tiredness)
- ☐ Irregular bleeding
- ☐ Indigestion
- ☐ Nausea
- ☐ Backache
- ☐ I did not purchase
- ☐ Other, please specify

24.a. If you selected Other, please specify:

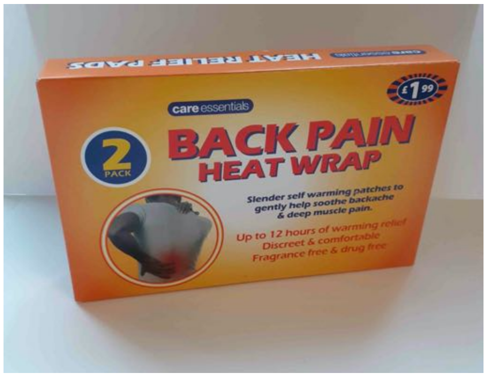

25. **Wheat bags, heat-pads or hot water bottles**

- ☐ Bloating
- ☐ Abdominal Pain (Tummy Pain)
- ☐ Change in urination habit
- ☐ Change in bowel habit
- ☐ Change in appetite
- ☐ Fatigue (Tiredness)
- ☐ Irregular bleeding
- ☐ Indigestion
- ☐ Backache
- ☐ Nausea
- ☐ I did not purchase
- ☐ Other, please specify

25.a. If you selected Other, please specify:

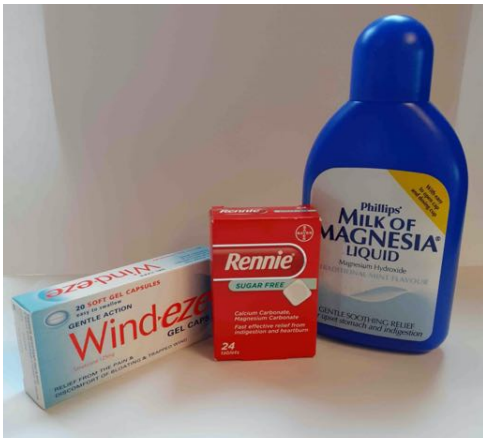

26. **Trapped Wind Product.** There are two types of this product. One type refers to after food, indigestion, upset stomach or heartburn mentioning discomfort. A second type refers to similar ailments but also mentions cramps or stomach pain as well. Please select if you purchased either of these types.

- ☐ Bloating
- ☐ Abdominal Pain (Tummy Pain)
- ☐ Change in urination habit
- ☐ Change in bowel habit
- ☐ Change in appetite
- ☐ Fatigue (Tiredness)
- ☐ Irregular Bleeding
- ☐ Indigestion
- ☐ Backache
- ☐ Nausea
- ☐ I did not purchase
- ☐ Other

26.a. If you selected Other, please specify:

[ ]

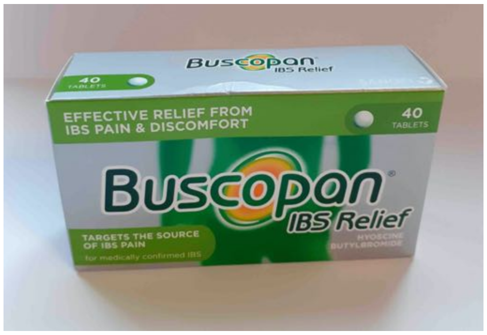

27. **IBS (Irritable bowel syndrome) Product.** Product packaging often refers to abdominal pain, spasms, painful cramps or stomach pain, may include relief from diarrhoea and constipation

- ☐ Bloating
- ☐ Abdominal Pain (Tummy Pain)
- ☐ Change in urination
- ☐ Change in bowel habit
- ☐ Change in appetite
- ☐ Fatigue (Tiredness)
- ☐ Irregular Bleeding
- ☐ Indigestion
- ☐ Backache
- ☐ Nausea
- ☐ I did not purchase
- ☐ Other

27.a. If you selected Other, please specify:

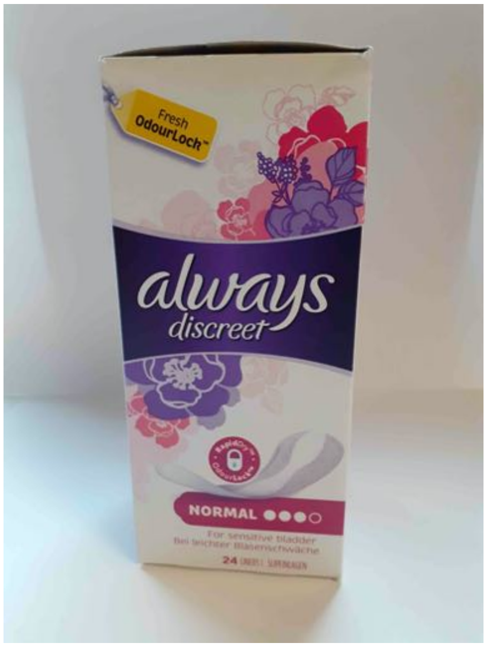

28. Incontinence or period products including pads, pants and tampons

- [ ] Bloating
- [ ] Abdominal Pain (Tummy Pain)
- [ ] Change in urination habit
- [ ] Change in bowel habit
- [ ] Change in appetite
- [ ] Fatigue (Tiredness)
- [ ] Irregular bleeding
- [ ] Indigestion
- [ ] Backache
- [ ] I did not purchase
- [ ] Nausea
- [ ] Other, please specify

28.a. If you selected Other, please specify:

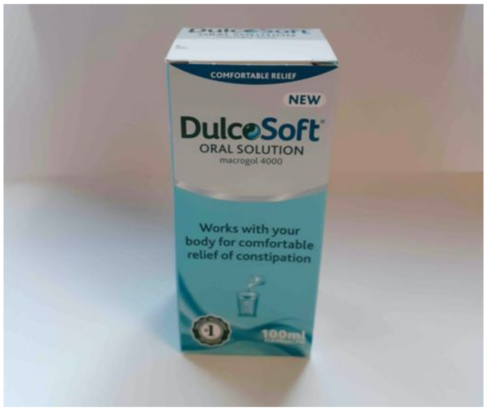

29. **Constipation Product**

- ☐ Bloating
- ☐ Abdominal Pain (Tummy Pain)
- ☐ Change in urination habit
- ☐ Change in bowel habit
- ☐ Change in appetite
- ☐ Fatigue (Tiredness)
- ☐ Irregular bleeding
- ☐ Indigestion
- ☐ Backache
- ☐ Nausea
- ☐ I did not purchase
- ☐ Other, please specify

29.a. If you selected Other, please specify:

____

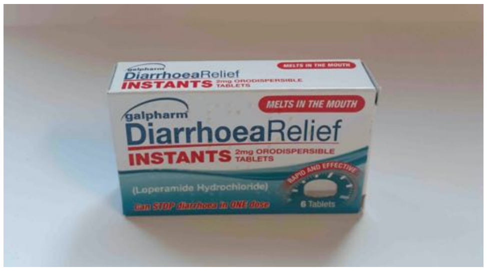

30. **Diarrhoea Product**

- [ ] Bloating
- [ ] Abdominal Pain (Tummy Pain)
- [ ] Change in urination habit
- [ ] Change in bowel habit
- [ ] Change in appetite
- [ ] Fatigue (Tiredness)
- [ ] Irregular bleeding
- [ ] Indigestion
- [ ] Backache
- [ ] Nausea
- [ ] I did not purchase
- [ ] Other, please specify

30.a. If you selected Other, please specify:

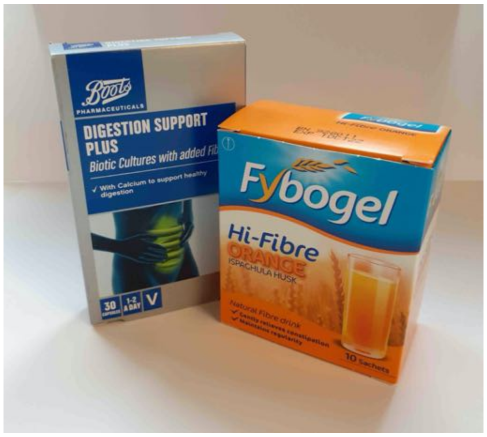

31. **Gut health products**. Including probiotics digestive enzymes, syrup of fig or fibre supplements to encourage healthy bowel habits

- [ ] Bloating
- [ ] Abdominal Pain (Tummy Pain)
- [ ] Change in urination habit

- [ ] Change in bowel habit
- [ ] Change in appetite
- [ ] Fatigue (Tiredness)
- [ ] Irregular bleeding
- [ ] Indigestion
- [ ] Backache
- [ ] Nausea
- [ ] I did not purchase
- [ ] Other, please specify

31.a. If you selected Other, please specify:

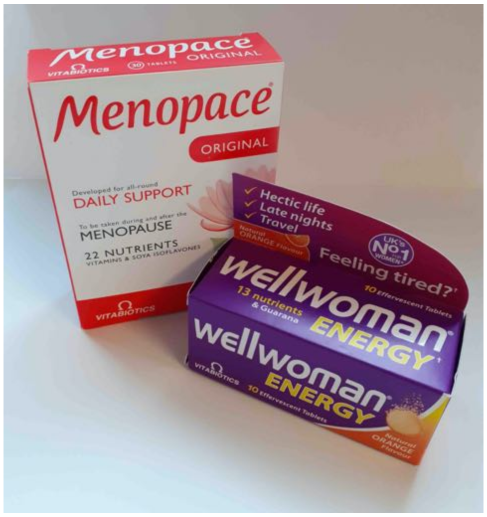

32. **Vitamins.** Often to increase energy or wellbeing including but not limited to iron tablets, supplements targeted at menopause.

- [ ] Bloating
- [ ] Abdominal Pain (Tummy Pain)
- [ ] Change in bowel habit
- [ ] Change in appetite
- [ ] Indigestion
- [ ] Nausea

- [ ] Change in urination habit
- [ ] Fatigue (Tiredness)
- [ ] Irregular bleeding
- [ ] Backache
- [ ] I did not purchase
- [ ] Other, please specify

32.a. If you selected Other, please specify:

____

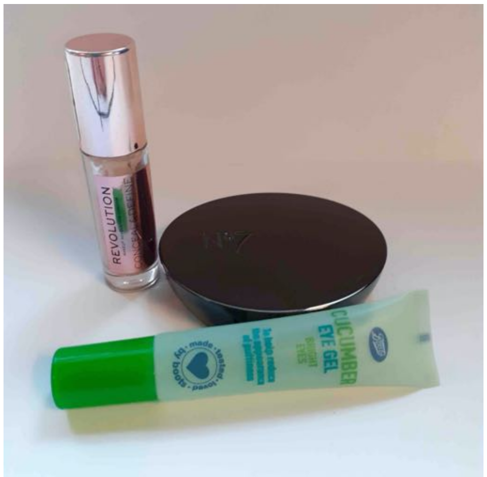

33. **Under eye cream and concealer products**

- [ ] Bloating
- [ ] Abdominal Pain (Tummy Pain)
- [ ] Change in bowel habit
- [ ] Change in urination habit
- [ ] Change in appetite
- [ ] Fatigue (Tiredness)
- [ ] Irregular Bleeding
- [ ] Indigestion
- [ ] Nausea
- [ ] Backache
- [ ] I did not purchase

☐ Other, please specify

33.a. If you selected Other, please specify:

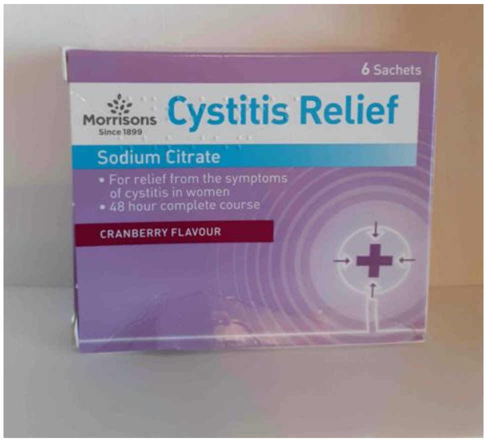

34. **Cystitis relief products**

☐ Bloating
☐ Abdominal Pain (Tummy Pain)
☐ Change in urination habit
☐ Change in bowel habit
☐ Change in appetite
☐ Fatigue (Tiredness)
☐ Irregular bleeding
☐ Indigestion
☐ Backache
☐ Nausea
☐ I did not purchase
☐ Other, please specify

34.a. If you selected Other, please specify:

35. Did you purchase items related to your health problems which were not mentioned above?

- Yes
- No

35.a. If yes, please state which items, and for which health problem:

Impact of items bought

The following section asks questions about your use of the products you bought. Please answer as best as you can remember. If you do not recall just move onto the next question.

If you did not purchase the product type in question move onto the next section.

Pain Relief

If you did not use pain relief, please skip to question 34 about abdominal health products.

36. Please use the table below to show how long the pain relief product worked for. The table allows for you to rate alternative pain relief products that you may have bought.

Please don't select more than 1 answer(s) per row.

	Pain relief did not work	A few hours	12-24 hours	A few days	1 week	2 weeks	1 month	Longer than 1 month
First Pain Relief Product	☐	☐	☐	☐	☐	☐	☐	☐
Alternative 1	☐	☐	☐	☐	☐	☐	☐	☐
Alternative 2	☐	☐	☐	☐	☐	☐	☐	☐
Alternative 3	☐	☐	☐	☐	☐	☐	☐	☐
Alternative 4	☐	☐	☐	☐	☐	☐	☐	☐

36.a. Please use this box to expand on how effective pain relief products were.

37. If the pain relief you bought reduced the pain did you buy it again?

○ Pain relief did not reduce the pain

- Yes - I bought it again
- No - I did not buy it again

37.a. If no why not?

38. If your pain increased did you change the amount of pain relief you bought?

- 1 - My pain did not increase
- 2 - When my pain increased I increased the amount of pain relief I bought
- 3 - When my pain increased I bought the same amount of pain relief
- 4 - When my pain increased I decreased the amount of pain relief I bought
- 5 - When my pain increased I no longer purchased pain relief

38.a. If you answered 4 or 5 please state why?:

Abdominal Health Products

If you did not use this product type, please skip to question 38 about vitamins and supplements.

39. Abdominal health products can take time to take effect. If you bought a product designed for trapped wind, indigestion, constipation, upset stomach or IBS how long did you wait to see if it would work?

- A few hours
- 12-24 hours
- A few days
- 1 week
- 2 weeks
- 3 weeks
- 1 month
- Longer than 1 month

39.a. Please use this box to expand on your answer about how long you waited to see if abdominal health products would take effect.

40. If you bought a product designed for trapped wind, indigestion, constipation, upset stomach, IBS please use the table below to show how long it worked for. The table allows for you to rate alternative abdominal products that you may have bought.

Please don't select more than 1 answer(s) per row.

	It did not work	A few hours	12-24 hours	A few days	1 week	2 weeks	1 month	Longer than 1 month
First Product	☐	☐	☐	☐	☐	☐	☐	☐
Alternative 1	☐	☐	☐	☐	☐	☐	☐	☐
Alternative 2	☐	☐	☐	☐	☐	☐	☐	☐
Alternative 3	☐	☐	☐	☐	☐	☐	☐	☐
Alternative 4	☐	☐	☐	☐	☐	☐	☐	☐

40.a. Please use this box to expand on how effective these adominal health products were.

41. If the abdominal health product designed for trapped wind, indigestion, constipation, upset stomach, IBS you bought reduced the health problem did you buy it again?

- The abdominal health products did not reduce the health problem
- Yes
- No

41.a. If no, why not?

42. If your health problem increased did you adjust the amount of abdominal health products designed for trapped wind, indigestion, constipation, upset stomach, IBS you bought?

- 1 - My health problem did not increase
- 2 - When my health problem increased I increased the amount I bought
- 3 - When my health problem increased I bought the same amount
- 4 - When my health problem increased I decreased the amount I bought
- 5 - When my health problem increased I no longer purchased the products

42.a. If you answered 4 or 5 please state why:

Vitamins and supplements

If you did not use these, please skip to question 42 about past shopping habits.

43. Vitamins and supplement health products can take time to take effect. If you bought this product type how long did you wait to see if it would work?

- A few hours
- 12-24 hours
- A few days
- 1 week
- 2 weeks
- 3 weeks
- 1 month
- Longer than 1 month

43.a. Please use this box to expand on your answer about how long you waited to see if vitamins or supplement products would take effect.

44. If you bought vitamins or supplement products please use the table below to show how long the product worked for. The table allows for you to rate alternative purchases that you may have bought.

Please don't select more than 1 answer(s) per row.

	Did not work	A few hours	12-24 hours	A few days	1 week	2 weeks	1 month	Longer than 1 month
First Vit/Sup Product	☐	☐	☐	☐	☐	☐	☐	☐
Alternative 1	☐	☐	☐	☐	☐	☐	☐	☐
Alternative 2	☐	☐	☐	☐	☐	☐	☐	☐
Alternative 3	☐	☐	☐	☐	☐	☐	☐	☐
Alternative 4	☐	☐	☐	☐	☐	☐	☐	☐

44.a. Please use this box to expand on how effective these vitamins or supplement health products were.

45. If the vitamins or supplements you bought reduced the health problem did you buy it again?

- ○ The vitamins or supplements did not reduce the health problem
- ○ Yes - I bought it again
- ○ No - I did not buy it again

45.a. If no, why not?

46. If your health problem increased did you increase the amount of vitamins or supplements you bought?

- 1 - My health problem did not increase
- 2 - When my health problem increased I increased the amount I bought
- 3 - When my health problem increased I bought the same amount
- 4 - When my health problem increased I decreased the amount I bought
- 5 - When my health problem increased I no longer purchased the vitamins or supplements

46.a. If you answered 4 or 5 please state why:

Past shopping habits

47. Before your health problems how often did you buy the following healthcare products?

Please don't select more than 1 answer(s) per row.

	Never	Rarely	Sometimes	Often	Always
Pain Relief	☐	☐	☐	☐	☐
Abdominal Products	☐	☐	☐	☐	☐
Vitamin / Supplements	☐	☐	☐	☐	☐

Loyalty card data

Only 6 questions to go...

The next questions are about loyalty card data. When customers use a 'loyalty' card at a shop it gives them points or rewards for buying items. It also collects information about what has been bought, by whom and when.

This shopping data could be used to give us more knowledge about the symptoms of ovarian cancer, if women are shopping in response to their health problems. For example the timeframes symptoms occur in, the order, and how women respond to them both physically and behaviourally.

This new knowledge from shopping data, gathered from loyalty cards, could help women and healthcare professionals recognise the signs of ovarian cancer earlier.

48. Please select which loyalty cards you have, and how often you use them:

Please don't select more than 1 answer(s) per row.

	Rarely	Sometimes	Often	Always
Boots Advantage Card	☐	☐	☐	☐
Tescos Clubcard	☐	☐	☐	☐
Nectar Card	☐	☐	☐	☐
Co-op Membership Card	☐	☐	☐	☐
Morrisons loyalty Card	☐	☐	☐	☐
Iceland Bonus Card	☐	☐	☐	☐
Waitrose Loyalty Card	☐	☐	☐	☐
M&S Sparks card	☐	☐	☐	☐
Superdrug Beauty Card	☐	☐	☐	☐

48.a. Other? Please state:

49. Would you consider giving your loyalty card data to help us analyse self-medication patterns for ovarian cancer? Please note, a part of this process would be anonymising your data, meaning you could not be individually identified from it.

- ○ Yes
- ○ No

49.a. If yes, please leave a contact email below to receive information about the project:

Demographics

We just need to ask a few last questions, as these are relevant to looking at how the risk of ovarian cancer varies.

50. Age?

[ ]

51. Are you a resident in the UK?

- ◯ Yes
- ◯ No

51.a. If yes, which part of the UK? Please select a region, or select Other - and input your nearest town or city.

- ◯ England - North East
- ◯ England - North West
- ◯ England - Yorkshire and The Humber
- ◯ England - East Midlands
- ◯ England - West Midlands
- ◯ England - East of England
- ◯ England - London
- ◯ England - South East
- ◯ England - South West
- ◯ Wales
- ◯ Scotland
- ◯ Northern Ireland
- ◯ Other - input your nearest town or city

51.a.i. If you selected Other, please specify:

52. What is your ethnic group? Choose one option that best describes your ethnic group or background

- White - English/Welsh/Scottish/Northern Irish/British
- White - Irish
- White - Gypsy or Irish Traveller
- White - Any other White background
- Mixed/Multiple ethnic groups - White and Black Caribbean
- Mixed/Multiple ethnic groups - White and Black African
- Mixed/Multiple ethnic groups - White and Asian
- Mixed/Multiple ethnic groups - Any other Mixed/Multiple ethnic background
- Asian/Asian British - Indian
- Asian/Asian British - Pakistani
- Asian/Asian British - Bangladeshi
- Asian/Asian British - Chinese
- Asian/Asian British - Any other Asian background
- Black/ African/Caribbean/Black British - African
- Black/ African/Caribbean/Black British - Caribbean
- Black/ African/Caribbean/Black British - Any other Black/African/Caribbean background
- Arab
- Prefer not to answer
- Other

52.a. If you selected Other, please specify:

Any other information

53. If there is anything else you would like to add that you feel relevant to this study, please use the box below to do so:

Final page

Thank you.

Every completed questionnaire will help us to inform future health research in this area.
Every added personal experience is valuable to us and helps us to improve our understanding. If you have any more questions about the research, please do not hesitate to get in touch.

Ovacome, who are assisting us with this survey, offer support for anyone affected by ovarian cancer. You can contact them via:

- 0800 008 7054 (open 10am-5pm Monday-Friday, 10am to 8pm on Tuesdays)
- Chat with them online https://www.ovacome.org.uk
- Or email support@ovacome.org.uk
