# Supplementary figures and images for: Using Shopping Data to Improve the Diagnosis of Ovarian Cancer: Computational Analysis of a Web-Based Survey
Source: JMIR Cancer. 2023 Mar 31;9:e37141. doi: 10.2196/37141 (PMC10131768; doi:10.2196/37141)

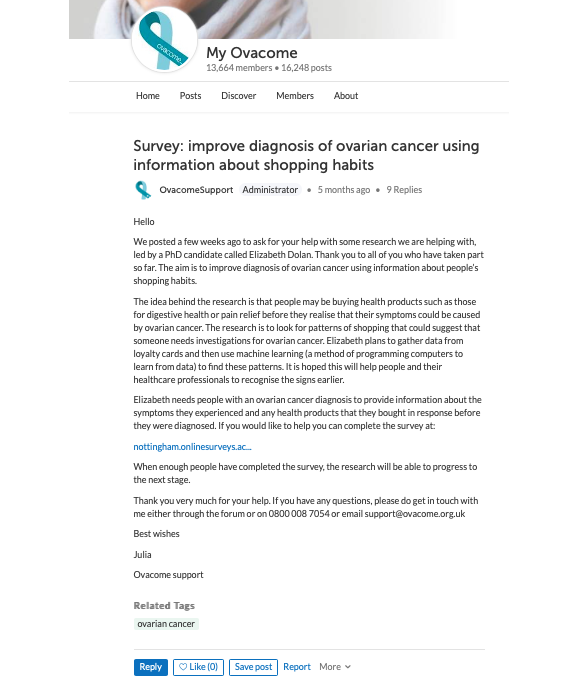

Supplement: Multimedia Appendix 2 [file cancer_v9i1e37141_app2.png]

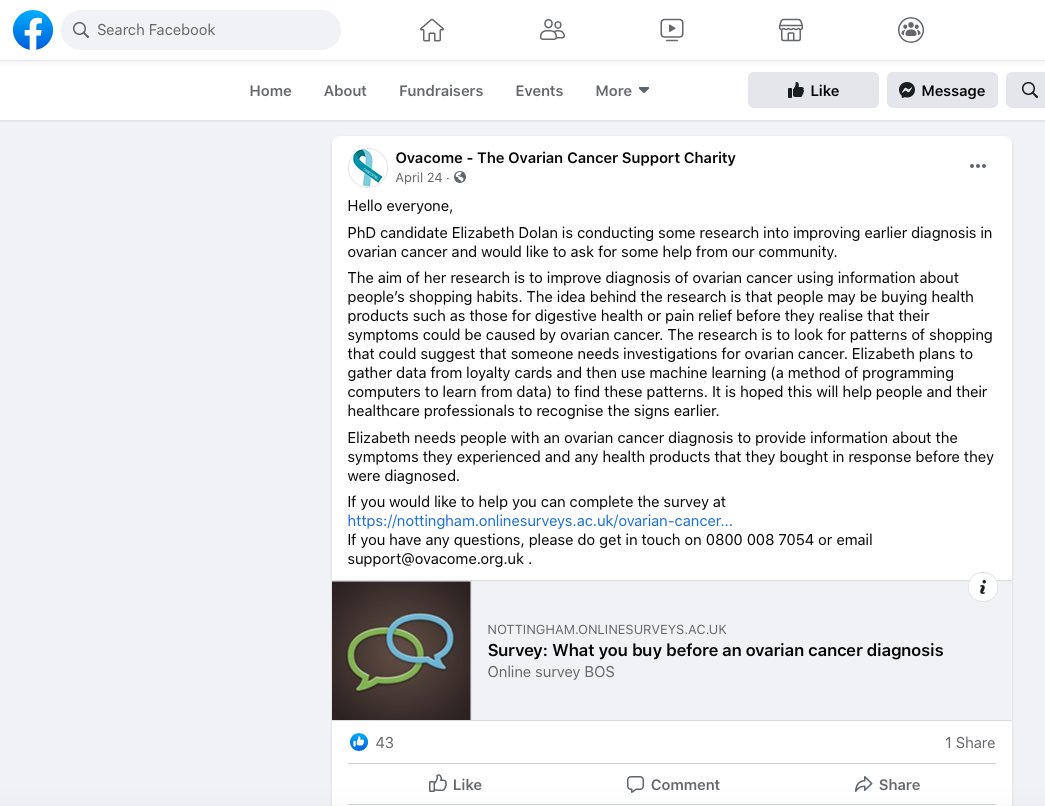

Supplement: Multimedia Appendix 3 [file cancer_v9i1e37141_app3.png]

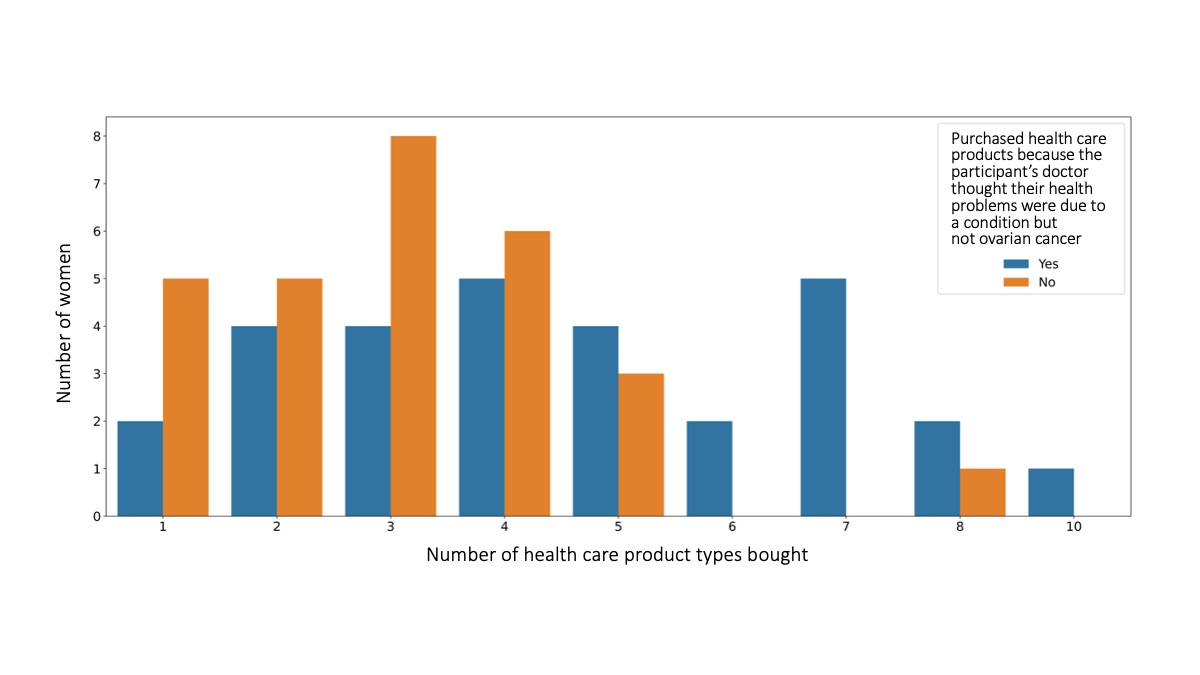

Supplement: Multimedia Appendix 5 [file cancer_v9i1e37141_app5.png]
