# Supplementary material for: Using Shopping Data to Improve the Diagnosis of Ovarian Cancer: Computational Analysis of a Web-Based Survey
Source: JMIR Cancer. 2023 Mar 31;9:e37141. doi: 10.2196/37141 (PMC10131768; doi:10.2196/37141)
Supplement: Multimedia Appendix 4 [file cancer_v9i1e37141_app4.pdf]

```
#!/usr/bin/env python
# coding: utf-8

# ### Diagnosing Disease with Shopping Data
#
```

```
# ## Using Shopping Data to Improve the Diagnosis of Ovarian Cancer:
Survey Study

# Considering the statistically significant results, participants’
duration of purchasing and amount of product types bought were
inputted into a random forest classification model, alongside
individual product types purchased.  Created using Scikit-Learn
library in Python, this was to explore if predictions determining
who had been misdiagnosed could be made from participants’ buying
patterns.

# In[208]:


#import python packages for analysis
#create dataframe from survey data
#change stage column to data type 'category', change diagnosis date
column data to 'datetime' data type

import pandas as pd
import numpy as np
import seaborn as sns
import matplotlib.pyplot as plt

df = pd.read_csv('OCSurveyData.csv',dtype={'OC Stage at
Diagnosis':'category'})

df['Diagnosis Date All'] = pd.to_datetime(df['Diagnosis Date All'])

#Change responses to "Bought Healthcare Products" to 'boolean'
datatype

df.loc[df['Bought Healthcare Products'] == 'I bought non-
prescription healthcare products in response to health problems I
was having before I was diagnosed with ovarian cancer.', 'Bought
non-prescription healthcare products'] = True
df.loc[df['Bought Healthcare Products'] == 'I did NOT buy non-
prescription healthcare products in response to health problems I
was having before I was diagnosed with ovarian cancer.', 'Bought
non-prescription healthcare products'] = False
```

```
# In[209]:


#shape and prepare data for "y" target (shopped due to misdiagnosis
or not) and "X" feature inputs  (duration of shopping, amount of
product types bought, product types purchased)

posGP = df[(df['Bought Healthcare Products Because Dr Suspected
Condition not OC'].notnull() & df['Bought Healthcare
Products'].str.contains('I bought non-prescription healthcare
products in response to health problems I was having before I was
diagnosed with ovarian cancer.'))]
posGP.shape


# In[210]:


posGP['Bought Healthcare Products Because Dr Suspected Condition not
OC'].shape


# In[211]:


#create list with 3 groups for duration of buying
num_in_threeGroups = []

a = 4
b = 8

for i in posGP['Duration of Buying Healthcare Products Prior to
Diagnosis Rank']:
    if i < a:
        num_in_threeGroups.append(1)
    elif i >= a and i < b:
        num_in_threeGroups.append(2)
    elif i == b:
        num_in_threeGroups.append(3)

print(num_in_threeGroups)


# In[212]:


len(num_in_threeGroups)


# In[213]:
```

```
#create dataframe from new list
groupNumber = pd.DataFrame(num_in_threeGroups, columns=["Group
Number"])

#describe new dataframe
groupNumber["Group Number"].describe()


# In[214]:


#find null entry
posGP['Duration of Buying Healthcare Products Prior to Diagnosis
Rank']


# In[215]:


#test dropping null entry
df2 = posGP.drop([10])


# In[216]:


df2['Duration of Buying Healthcare Products Prior to Diagnosis
Rank']


# In[217]:


#reshape data
groupRank = groupNumber.values.reshape(-1,1)


# In[218]:


#create dataframe with relevent columns from survey data on products
purchased
#including sum of bought items
dataShopping = df.iloc[:,[53,55,57,59,61,63,65,67,69,71,73,75]]
dataShopping = dataShopping.fillna(0).replace("I did not purchase",
0).astype(bool).astype(int)
dataShopping = dataShopping[(df['Bought Healthcare Products Because
Dr Suspected Condition not OC'].notnull() & df['Bought Healthcare
Products'].str.contains('I bought non-prescription healthcare
products in response to health problems I was having before I was
diagnosed with ovarian cancer.'))]
#sum products bought
dataShopping["No of Product Types Bought"]= dataShopping.sum(axis=1)
dataShopping.head()
```

```
# In[219]:


dataShopping.shape


# In[220]:


#drop entry with null value
dataShopping1 = dataShopping.drop([10])


# In[221]:


dataShopping1.shape


# In[222]:


# add shopping duration group to data in preparation for X inputs
dataShopping1['Duration of Buying'] = groupRank


# In[223]:


dataShopping1


# In[224]:


ds = dataShopping1[[ 'Duration of Buying','No of Product Types
Bought','Pain relief', 'Pain relief with codeine', 'Wheat bags,
heat-pads or hot water bottles','Trapped Wind Product',
                    'IBS Product','Incontinence or period products',
'Constipation Product', 'Gut health products', 'Vitamins',
                    'Under eye cream and concealer products']]
ds


# In[225]:


#import package to create dummy model for baseline
from sklearn import dummy

# assign X inputs and y target
```

```
X = ds

y = df2['Bought Healthcare Products Because Dr Suspected Condition
not OC'].replace("Yes", 1).replace("No", 0)

#-- create a dummy classifier as our baseline
dc = dummy.DummyClassifier()

#-- Fit the model on data
dc.fit(X, y)

#-- Obtain the score (performance) of our classifier
score = dc.score(X, y)

#-- print the results
print("Baseline Accuracy:", score)
print(len(y.values))


# In[226]:


#-- a confusion matrix for dummy classifier
from sklearn.metrics import confusion_matrix


# In[227]:


cm = confusion_matrix(y, dc.predict(X))

fig, ax = plt.subplots(figsize=(8, 8))
ax.imshow(cm)
ax.grid(False)
ax.xaxis.set(ticks=(0, 1), ticklabels=('Predicted 0s', 'Predicted
1s'))
ax.yaxis.set(ticks=(0, 1), ticklabels=('Actual 0s', 'Actual 1s'))
ax.set_ylim(1.5, -0.5)
for i in range(2):
    for j in range(2):
        ax.text(j, i, cm[i, j], ha='center', va='center',
color='red')
plt.show()


# In[228]:


#-- Add the required import for the new RF classifier
from sklearn.ensemble import RandomForestClassifier

#-- create classifier
rf = RandomForestClassifier()
```

```
# In[229]:


#import grid search
from sklearn.model_selection import GridSearchCV


# In[230]:


#create cross-validation grid search function

def rf_grid_search(X,y,nfolds):
    #create a dictionary of all values we want to test
    # define search space
    param_grid = dict()
    param_grid['n_estimators'] = [10, 100, 500]
    param_grid['max_features'] = [4,5,6,7,8]

    param_grid['max_depth'] = [5] #set to prevent overfitting on
small sample size
    param_grid['min_samples_leaf'] = [2] #set to prevent overfitting
on sample sample size

    # rf tree model
    rf_model=RandomForestClassifier()
    #use gridsearch to test all values
    rf_gscv = GridSearchCV(rf_model, param_grid, cv=nfolds)
    #fit model to data
    rf_gscv.fit(X, y)
    return rf_gscv.best_params_


# In[231]:


#run grid serach function with 10 folds
rf_grid_search(X_train, Y_train, 10)


# In[232]:


#Train model with parameters from gridsearch
rf = RandomForestClassifier(max_depth=5, max_features=4,
min_samples_leaf=2, n_estimators=100)


# In[261]:


#fit model to all data
rf.fit(X, y)
```

```
# In[262]:


cm = confusion_matrix(y, rf.predict(X))

fig, ax = plt.subplots(figsize=(8, 8))
ax.imshow(cm)
ax.grid(False)
ax.xaxis.set(ticks=(0, 1), ticklabels=('Predicted 0s', 'Predicted
1s'))
ax.yaxis.set(ticks=(0, 1), ticklabels=('Actual 0s', 'Actual 1s'))
ax.set_ylim(1.5, -0.5)
for i in range(2):
    for j in range(2):
        ax.text(j, i, cm[i, j], ha='center', va='center',
color='red')
plt.show()


# In[263]:


from sklearn.metrics import classification_report, confusion_matrix

# Step 4: Evaluate the model
p_pred = rf.predict_proba(X)
y_pred = rf.predict(X)
score_ = rf.score(X, y)
conf_m = confusion_matrix(y, y_pred)
report = classification_report(y, y_pred)


# In[264]:


score_


# In[265]:


print(classification_report(y, rf.predict(X)))


# In[266]:


rf.feature_importances_


# In[267]:
```

```
sorted_idx = rf.feature_importances_.argsort()
plt.barh(ds.columns[sorted_idx],
rf.feature_importances_[sorted_idx])
plt.xlabel("Random Forest Feature Importance")

# Save chart:
plt.savefig('feature_import.eps', format='eps', bbox_inches="tight")
("")


# In[ ]:


# Nested cross validation for RF accuracy score, random_state 42

from sklearn.model_selection import KFold
from sklearn.model_selection import cross_val_score

# configure the cross-validation procedure
cv_inner = KFold(n_splits=10, shuffle=True, random_state=42)
# define the model
rf_model = RandomForestClassifier()
# define search space
param_grid = dict()
param_grid['n_estimators'] = [10, 100, 500]
param_grid['max_features'] = [4,5,6,7,8]
param_grid['max_depth'] = [5]
param_grid['min_samples_leaf'] = [2]

# define search
search = GridSearchCV(rf_model, param_grid, scoring='accuracy',
n_jobs=1, cv=cv_inner, refit=True)
# configure the cross-validation procedure
cv_outer = KFold(n_splits=10, shuffle=True, random_state=42)
# execute the nested cross-validation
scores_accuracy = cross_val_score(search, X, y, scoring='accuracy',
cv=cv_outer, n_jobs=-1)


# In[ ]:


scores_accuracy


# In[ ]:


scores_accuracy.mean()


# In[ ]:
```

```
scores_accuracy.std()


# In[ ]:


# Nested cross validation for RF precision score, random_state 42

# configure the cross-validation procedure
cv_inner = KFold(n_splits=10, shuffle=True, random_state=42)
# define the model
rf_model = RandomForestClassifier()
# define search space
param_grid = dict()
param_grid['n_estimators'] = [10, 100, 500]
param_grid['max_features'] = [4,5,6,7,8]
param_grid['max_depth'] = [5]
param_grid['min_samples_leaf'] = [2]

# define search
search = GridSearchCV(rf_model, param_grid, scoring='precision',
n_jobs=1, cv=cv_inner, refit=True)
# configure the cross-validation procedure
cv_outer = KFold(n_splits=10, shuffle=True, random_state=42)
# execute the nested cross-validation
scores_precision = cross_val_score(search, X, y,
scoring='precision', cv=cv_outer, n_jobs=-1)


# In[ ]:


scores_precision


# In[ ]:


scores_precision.mean()


# In[ ]:


scores_precision.std()


# In[59]:


# Nested cross validation for RF recall score, random_state 42

# configure the cross-validation procedure
```

```
cv_inner = KFold(n_splits=10, shuffle=True, random_state=42)
# define the model
rf_model = RandomForestClassifier()
# define search space
param_grid = dict()
param_grid['n_estimators'] = [10, 100, 500]
param_grid['max_features'] = [4,5,6,7,8]
param_grid['max_depth'] = [5]
param_grid['min_samples_leaf'] = [2]

# define search
search = GridSearchCV(rf_model, param_grid, scoring='recall',
n_jobs=1, cv=cv_inner, refit=True)
# configure the cross-validation procedure
cv_outer = KFold(n_splits=10, shuffle=True, random_state=42)
# execute the nested cross-validation
scores_recall = cross_val_score(search, X, y, scoring='recall',
cv=cv_outer, n_jobs=-1)


# In[60]:


scores_recall


# In[61]:


scores_recall.mean()


# In[63]:


scores_recall.std()


# In[ ]:
```
